# Risk Assessment of Phthalate Esters in Baiyangdian Lake and Typical Rivers in China

**DOI:** 10.3390/toxics11020180

**Published:** 2023-02-15

**Authors:** Yin Hou, Mengchen Tu, Cheng Li, Xinyu Liu, Jing Wang, Chao Wei, Xin Zheng, Yihong Wu

**Affiliations:** 1 State Key Laboratory of Environmental Criteria and Risk Assessment, Chinese Research Academy of Environmental Sciences, Beijing 100012, China; 2 College of Marine Ecology and Environment, Shanghai Ocean University, Shanghai 201306, China; 3 Institute of Green Development, Hebei Provincial Academy of Environmental Sciences, Shijiazhuang 050037, China

**Keywords:** phthalate esters, reproductive toxicity, aquatic life criteria, sediment quality criteria, ecological risk assessment

## Abstract

Phthalate esters (PAEs) are frequently tracked in water environments worldwide. As a typical class of endocrine disruptor chemicals (EDCs), PAEs posed adverse effects on aquatic organisms at low concentration. Thus, they have attracted wide attention in recent years. In the present study, the concentrations of seven typical PAEs from 30 sampling sites in Baiyangdian Lake were measured, and the environmental exposure data of PAEs were gathered in typical rivers in China. Then, based on the aquatic life criteria (ALCs) derived from the reproductive toxicity data of aquatic organisms, two risk assessment methods, including hazard quotient (HQ) and probabilistic ecological risk assessment (PERA), were adopted to evaluate the ecological risks of PAEs in water. The sediment quality criteria (SQCs) of DEHP, DBP, BBP, DIBP and DEP were deduced based on the equilibrium partitioning method. Combined with the gathered environmental exposure data of seven PAEs in sediments from typical rivers in China, the ecological risk assessments of five PAEs in sediment were conducted only by the HQ method. The results of ecological risk assessment showed that in terms of water, DBP and DIBP posed low risk, while the risk of DEHP in Baiyangdian Lake cannot be ignored and should receive attention. In typical rivers in China, BBP and DEP posed no risk, while DIBP and DBP posed potential risk. Meanwhile, DEHP posed a high ecological risk. As far as sediment is concerned, DBP posed a high risk in some typical rivers in China, and the other rivers had medium risk. DEHP posed a high risk only in a few rivers and low to medium risk in others. This study provides an important reference for the protection of aquatic organisms and the risk management of PAEs in China.

## 1. Introduction

Phthalate esters (PAEs), also known as phenolic acid ester, are intensively applied in plastic products as plasticizers to improve their elasticity, toughness and durability. In addition, PAEs are widely used in the production of paints, pesticides, fertilizers, herbicides, pesticides, solvents and cosmetics. It is reported that in 2014, PAEs accounted for 70% of the 8.4 million tons of plasticizers produced worldwide, and they will continue to grow at a high rate of 3.9% over the next 5 years [1]. Li et al. found that the total amount of PAEs consumed in China reached 2.2 million tons in 2011 alone [2]. Since PAEs do not chemically bond with polymer molecules of plastic products, they are easy to dissociate from the above products and leach into the environment under certain conditions [3,4]. After the mass production, use and disposal of substances containing PAEs, their pollution is distributed in various environments around the world, including air, soil, sediment, landfill leachate, urban runoff and natural water [5,6,7]. PAEs mainly enter the water environment through the discharge of domestic sewage and industrial wastewater, the input of surface runoff from agricultural and urban areas, and the dry and wet settlement of the atmosphere [8,9,10]. Then, they settle in the bottom sediment with particulate matter and accumulate continuously. Therefore, the concentrations of PAEs are relatively high in the sediments [11]. With the migration and transformation of PAEs in different environments and the long-distance transport in the global scale, PAEs has become a kind of global organic pollutant that is widely detected in rivers, lakes, reservoirs and their sediments around the world. For example, Vietnamese scholar Le et al. conducted a survey in six lakes including Tien Quang Lake in Vietnam and found that the highest concentration of PAEs reached 127 µg/L [12]. The highest concentration of PAEs was 4.64 µg/L in the Kaveri River in India [13]. Liu et al. investigated di (2-ethylhexyl) phthalate (DEHP) in 31 surface waters of seven major river basins in China, and the results showed that the concentration of DEHP ranged from 0.01000 to 2634 µg/L, among which the pollution of PAEs in Xuanwu Lake and Anshan Urban Rivers were the most serious, and the concentration was as high as 1.3 × $10^{3}$ and 1.3 × $10^{4}$ µg/L, respectively [14]. Li et al. studied the PAEs in the sediments of urban rivers in northeast China and found that the total concentration of PAEs in the sediment of Xi River, a tributary of Liao River, ranged from 22.4 to 369 μg/g dw, and the pollution of PAEs in the sediment was more serious [2]. Since PAEs are difficult to be degraded and have a high degree of bioaccumulation, they can produce reproductive, developmental and neurotoxic effects on aquatic organisms after entering the aquatic environment [15]. Otherwise, they can enter the human body through different exposure pathways, causing reproductive and developmental toxicity and even carcinogenesis [16,17]. The United States Environmental Protection Agency (EPA) has listed six PAEs including dimethyl phthalate (DMP), diethyl phthalate (DEP), di-n-butyl phthalate (DBP), butyl-benzyl phthalate (BBP), di-n-octyl phthalate (DOP) and DEHP in the 129 environmental priority pollutants field [18]. China also listed DBP, DMP and DOP as environmental priority pollutants [19].

ALC refers to the maximum concentration of pollutants that do not cause short-term or long-term adverse effects and harm to aquatic organisms [20]. Species sensitivity distribution (SSD) is an important extrapolation method, which can be used to derive ALCs from toxicological data of pollutants and extrapolate the corresponding pollutants concentration (HCx) for a target percentage of species affected [21]. Lethal effect is usually taken as the toxicity endpoint to construct SSD when deriving ALCs of conventional pollutants such as ammonia nitrogen and heavy metals, while PAEs as a typical class of EDCs that generally affect the reproduction of organisms at low concentrations [22,23]. Thus, ALCs derived from PAEs based on a lethal toxicity endpoint cannot provide sufficient protection for aquatic organisms. Previous studies have shown that reproductive toxicity endpoints were the most sensitive for EDCs [24,25], and reproductive toxicity includes fertility, fertilization rate, hatchability, gonadal index that lasts for multiple generations, and the synthesis of vitellogenin (VTG) [26]. Therefore, reproduction was the most suitable endpoint for deriving the ALCs of PAEs.

For the derivation of PAEs sediment quality criteria (SQC), considering that there are few studies on sediment benthic and the toxicity data are not enough to construct SSD, the equilibrium partitioning method recommended in the European Union Technical Guidelines for Risk Assessment (TGD) is referred. The equilibrium distribution method is applicable to nonionic organic compounds with lg$K_{ow}$ (logarithm of octanol–water partition coefficient) > 3. This method is based on the following assumptions: (1) the organisms living in the sediment environment and in water have the same sensitivity to pollutants; (2) the concentrations of pollutants in sediment, interstitial water and benthic organisms are in thermodynamic equilibrium, and the equilibrium partition coefficient can be used to predict the concentration of pollutants in any phase.

Ecological risk assessment (ERA) refers to the assessment of the possibility of adverse ecological consequences after the ecosystem is affected by one or more stress factors [27]. The hazard quotient (HQ) is a point estimate method of ecological risk, which has the advantages of simplicity and low data requirement. However, the magnitude and probability of occurrence of ecological risk of pollutants cannot be evaluated by the HQ method, and it is only applicable to preliminary risk assessment [28,29]. Probabilistic ecological risk assessment (PERA) is a higher level ecological risk assessment method [28]. Joint probability curve (JPC) is one of the commonly used methods of probabilistic risk assessment. This curve is fitted based on the toxicity data and exposure data, which reflects the probability that the exposure concentration exceeds the corresponding critical concentration at different damage levels, that is, the risk degree of pollutants in the environment to aquatic organisms at different damage levels, and the probability of adverse effects concentration (HCx) of pollutants in water on a target percentage of aquatic organisms can be obtained [25,30]. The closer the joint probability curve is to the x axis, the less the aquatic organisms are affected by pollutants, and the less the occurrence probability of ecological risk of water. Each point on the JPC represents the probability that a target percentage of organisms being affected (events) will occur in the target water (the evaluation object).

As a global organic pollutant, PAEs are clearly harmful to the environment. However, a large number of PAEs are still used every year and are constantly released into the environment from production and living activities, affecting and endangering biosecurity and even ecosystem stability. Baiyangdian Lake is the largest freshwater lake in the North China Plain and Xiongan; the new area attaches great importance to the water environment. This study conducted a comprehensive field investigation on Baiyangdian Lake and assessed the ecological risk of PAEs, which can effectively control the pollution of PAEs and provide data support and a theoretical basis for the formulation of water quality standards and the prevention and control of PAEs pollution in the future.

## 2. Material and Methods

### 2.1. Solvents and Chemical Standards

DEHP, DBP, BBP, DEP, DMP, DOP and DIBP were investigated in this work, and their characteristics are summarized in Table S1. Methanol (pesticide grade) was purchased from J.T. Baker Co. USA. Hydrochloric acid, ethyl acetate and dichloromethane (pesticide grade) were purchased from Bailingwei Company (Beijing, China). A mixed standard solution of the 6 PAEs was used. Benzyl benzoate (BBZ) was used as the internal standard, and these substances were all obtained from Sigma-Aldrich (St. Louis, MO, USA).

### 2.2. Sample Collection and Preparation

**Figure 1:**
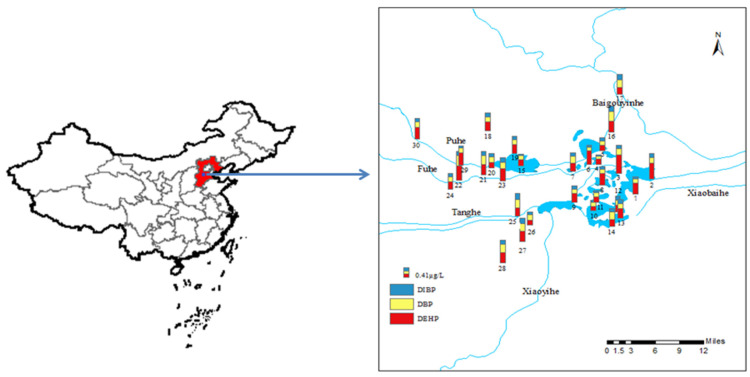
Location of sampling sites and PAEs concentration in Baiyangdian Lake.

Sampling sites were set based on the Baiyangdian Lake entrance, the interchange, the farmland area, the living area, etc., and a total of 15 sites were arranged on 8 rivers which entering the Baiyangdian Lake. Then, according to the national control monitoring sites and 40 lakes in Baiyangdian Lake, 15 sites were arranged, so 30 sites in total were arranged in Baiyangdian Lake. The sites distribution and the concentration of PAEs at each site are shown in Figure 1. In April 2019, water samples were collected in 2 L brown glass bottles. The sample bottles were cleaned with tap water, distilled water and methanol, respectively, in the laboratory for 3 times in advance, and then, they were moistened and washed with on-site water 3 times. After the samples were collected, the pH was adjusted to 2.0 with 4 mol·L^−1^ hydrochloric acid (to inhibit microbial activity), and the samples were stored in a refrigerator and transported back to the laboratory for pretreatment within 24 h.

The preparation of the water samples prepared for gas chromatography mass spectrometry (GC-MS) was performed. Firstly, 0.45 μm glass fiber filters (GF/F, Whatman, UK) were pre-burned for 4 h in a muffle furnace at 400 °C, and then, 1 L water samples were filtered using the filters. Secondly, solid phase extraction was performed. Before concentrating and enriching the sample, C18 solid phase extraction columns (BOJIN SPE Column C18) were activated with 5 mL dichloromethane, 5 mL ethyl acetate, 1 mL methanol and 10 mL ultrapure water, respectively. The controlled flow rate of the C18 SPE Columns was 3 mL·min^−1^. The sample bottles were cleaned with 5 mL ethyl acetate and entered into the collection bottle through the C18 SPE columns; then, they were cleaned with 5 mL dichloromethane and entered into the same collection bottle through the C18 SPE columns. Finally, the sample extracts were blown to nearly dry with nitrogen at 45 °C and reconstituted to 1 mL by adding 5 μL BBZ and ethyl acetate, after which they were sealed and stored at 4 °C before GC/MS analysis. After extraction, the C18 SPE columns were rinsed with 10 mL ultrapure water and then blown with nitrogen for 5 min to remove water.

### 2.3. Chemical Analysis

The analysis of sample extracts was conducted by a GC-MS system (Aglient7890-5975 C) with a DB-5 capillary column (30 m × 0.25 mm × 0.25 μm) (Agilent Technologies, Santa Clara, CA, USA). The oven temperature starts at 70 °C and was maintained for 2 min; then, it increased to 180 °C at 40 °C/min and was maintained for 2 min, and finally, it increased to 280 °C at 10 °C/min for 2 min. The samples were injected in splitless mode with helium as the carrier gas at a flow rate of 1 mL/min. The transfer line, quadrupole and ion source were 290 °C, 150 °C and 230 °C, respectively. The system was operated in electron impact (EI) and scan modes.

The instrument required for testing should be recalibrated daily according to the calibration standard before use, and the sample should also be processed with the program blank in order to improve the accuracy of data [31]. The recoveries of the PAEs in the water samples were 85.60–116.7%. The PAEs were calibrated with BBZ and recoveries of 94.8% in the water. The detection limits (signal to noise ratio = 3) for the PAEs were 0.060–0.84 ng/L for water.

### 2.4. Collection of Data

(1) Toxicity data

The toxicity data of PAEs were obtained from the ECOTOX database (https://cfpub.epa.gov/ecotox, accessed on 1 September 2021), the Web of Science database, the CNKI database and other published literature. However, the screening of toxicity data was only based on reproductive-related endpoints, such as fertility, fertilization rate, hatchability, gonadal index that lasts for multiple generations, and the synthesis of vitellogenin (VTG). The screening principles for toxicity data must be followed. No observed effect concentration (NOEC) was selected as the preferred toxicity endpoint data. The maximum acceptable toxicant concentration (MATC) was used when NOEC was not available. If neither NOEC nor MATC were available, the lowest observed effect concentration (LOEC) or 10% effective concentration (EC_10_) value was used. The toxicity data of four PAEs are shown in Tables S2 and S3. In order to avoid possible data bias due to similar species and observed duration, the geometric mean value was adopted [32].

(2) Exposure data

In order to compare with the PAEs pollution in Baiyangdian Lake, the exposure data of PAEs in freshwater such as rivers, lakes and reservoirs and their sediments in China were also collected from the Web of Science database, the CNKI database and other published literature from 2007 to 2019.

### 2.5. Deriving of ALCs and SQCs for PAEs

(1) Deriving of ALCs

To construct SSDs based on the method of technical guideline [33].

For the chronic toxicity data collected, the MATC of reproductive toxicity of a species was calculated by the following formula:

(1) $${\mathrm{MATC}}_{i,z}=\sqrt{{\mathrm{NOEC}}_{i,z}\times {\mathrm{LOEC}}_{i,z}}$$

where:

MATC = maximum acceptable toxicant concentration, mg/L or μg/L;

NOEC = no observed effect concentration, mg/L or μg/L;

LOEC = lowest observed effect concentration, mg/L or μg/L;

i = a certain species, dimensionless;

z = a toxic effect, dimensionless.

Then, chronic toxicity data (MATC, EC10, EC_20_, NOEC, LOEC, EC_50_ and LC_50_) were used as growth or reproductive CTV, and LC50 was used as survival CTV, which were substituted into Formula (3) to calculate the growth CVE, reproductive CVE and survival CVE of each species. This study calculated the reproductive CVE of each species.

(2) $${\mathrm{CVE}}_{i,j}=\sqrt[{n}]{{\mathrm{CTV}}_{i,j,1}\times {\mathrm{CTV}}_{i,j,2}\times \ldots \times {\mathrm{CTV}}_{i,j,n}}$$

where

CVE = chronic value for the same effect, μg/L or mg/L;

i = a certain species, dimensionless;

j = types of chronic toxic effects, generally classified as growth, survival and reproduction, dimensionless; 

CTV = chronic toxicity value, mg/L or μg/L;

n = the number of CTV.

Arrange lgCVE from small to large, determine its rank R, and adopt Formula (3) to calculate the chronic cumulative frequency $F_{R}$ of species.

(3) $$F_{R}=\frac{\sum_{1}^{R}f}{N+1}\times 100\%$$

where

$F_{R}$ = cumulative frequency, %;

R = the rank of toxicity value, dimensionless;

f = frequency, refers to the number of species corresponding to the rank of toxicity value;

N = the sum of all the frequencies.

**Figure 2:**
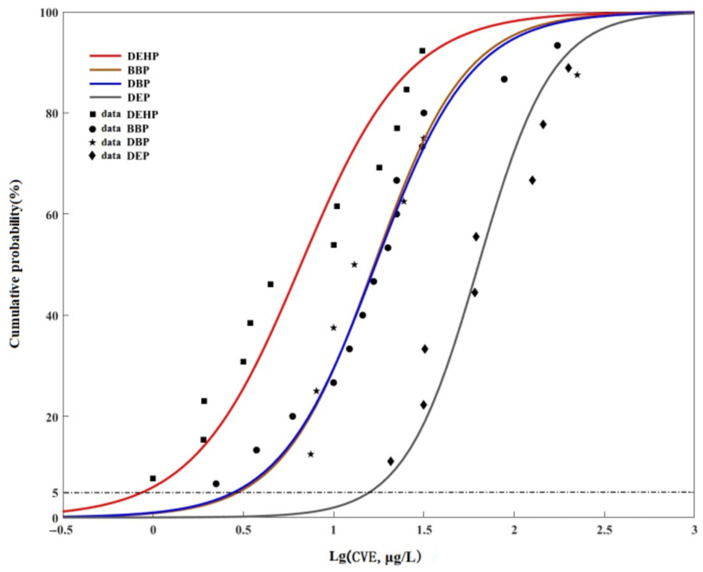
Species sensitivity distribution curves of PAEs.

In addition, with lgCVE as independent variable x, and the corresponding cumulative frequency $F_{R}$ as dependent variable y, the SSD model was fitted by the log-logistic distribution, which was a good-fitting model for SSD (Figure 2) [21]. The hazardous concentration for 5% species affected (HC_5_) was calculated from the SSD curves.

Then, ALC is calculated as the HC_5_ divided by an assessment factor of 3, because the number of species included in effective toxicity data was less than or equal to 15. The results are shown in Table 1. Since the amount of toxicity data of DIBP does not meet the minimum data requirements of SSD, the ALC of DIBP obtained by the assessment factor method was 0.90000 μg/L, and the AF value of 1000 was taken into account as the “worst case” in the ecological risk assessment [34].

(2) Deriving of SQCs

The SQCs measured by wet mass were derived using the equilibrium partitioning method [35], and the formulas are as follows:

(4) $${\mathrm{SQC}}_{\mathrm{wet}\;\mathrm{mass}}=\frac{K_{\mathrm{susp}\text{-}\mathrm{water}}}{{\mathrm{RHO}}_{\mathrm{susp}}}\times \mathrm{ALC}\times 1000$$

(5) $${\mathrm{RHO}}_{\mathrm{susp}}={\mathrm{Fsolid}}_{\mathrm{susp}}\times {\mathrm{RHO}}_{\mathrm{solid}}+{\mathrm{Fwater}}_{\mathrm{susp}}\times {\mathrm{RHO}}_{\mathrm{water}}$$

(6) $$K_{\mathrm{susp}\text{-}\mathrm{water}}={\mathrm{Fwater}}_{\mathrm{susp}}+{\mathrm{Fsolid}}_{\mathrm{susp}}\times \frac{{\mathrm{Foc}}_{\mathrm{susp}}\times K_{\mathrm{oc}}}{1000}\times {\mathrm{RHO}}_{\mathrm{solid}}$$

where

SQC_wet mass_ = sediment quality criteria measured by wet mass, mg/kg;

${\mathrm{RHO}}_{\mathrm{susp}}$ = bulk density of (wet) suspended matter, kg/m^3^;

$K_{\mathrm{susp}\text{-}\mathrm{water}}$ = partitioning coefficient in suspended matter and water, m^3^/m^3^;

Fsolid_susp_ = φ (solid matter) in suspended matter, which was defined as 0.1 m^3^/m^3^;

${\mathrm{RHO}}_{\mathrm{solid}}$ = bulk density of solid matter, which was defined as 2500 kg/m^3^;

Fwater_susp_ = φ(H_2_O) in suspended matter, which was defined as 0.9 m^3^/m^3^;

${\mathrm{RHO}}_{\mathrm{water}}$ = water density, which was defined as 1000 kg/m^3^;

${\mathrm{Foc}}_{\mathrm{susp}}$ = organic carbon fraction of solid matter in suspended matter, 0.1 kg/kg in this study;

Koc = pollutant organic carbon—water partitioning coefficient, L/kg, that is, the ratio of the concentration of PAEs in sediment organic carbon and water.

### 2.6. Ecological Risk Assessment

In this study, two ecological risk assessment methods were used to evaluate the ecological risks of PAEs in Baiyangdian Lake and typical rivers in China, including the low-level HQ method and the high-level PERA method. However, only the HQ method was used to evaluate the ecological risks of PAEs in sediments from typical rivers in China.

(1) Hazard quotient (HQ)

HQ was calculated by the Equation (7):

(7) $$\mathrm{HQ}=\frac{\mathrm{EEC}}{\mathrm{ALC}}\;\mathrm{or}\;\frac{\mathrm{EEC}}{\mathrm{SQC}}$$

where EEC = environmental exposure concentration, μg/L or mg/kg.

The value obtained by the HQ method can be classified into the following 4 levels to evaluate the ecological risk [36]:

HQ ≤ 0.1, no risk;

HQ = 0.1–1.0, there is low risk;

HQ = 1.1–10, medium risk;

HQ ≥ 10, high risk.

The ecological risk assessments of PAEs in sediment also adopt the HQ method, but the risk classification is different from water: there was high risk when HQ > 1 (the lg$K_{\mathrm{ow}}$ of PAE congener was between 3 and 5) and high risk when HQ < 10 (lg$K_{\mathrm{ow}}$ > 5) [37]. For PAEs with lg$K_{\mathrm{ow}}$ < 3, their risk to aquatic organisms was not considered, because they are not easily adsorbed in sediment [34].

(2) Probabilistic ecological risk assessment (PERA)

Compared with the HQ method, PERA is an improved and higher ecological risk assessment method, because it can better describe the possibility that the concentration of a certain pollutant in water exceeds the toxic effect threshold and the risk of adverse effects [28]. Matlab software was used to draw JPCs to evaluate the ecological risks of PAEs. Firstly, log-normal distribution test was conducted on the exposure data of some PAEs in Baiyangdian Lake and typical rivers in China and the chronic toxicity data of aquatic organisms based on the endpoint of reproductive toxicity test. After that, the cumulative function of chronic toxicity data and the anti-cumulative function of PAEs exposure data were plotted to obtain the JPCs of PAEs [38,39].

## 3. Results

### 3.1. Occurrence and Composition of PAEs in Baiyangdian Lake

Through investigation and previous research [40], Baiyangdian Lake was divided into five functional areas: primitive area, tourism area, living area, breeding area and inflow area; each functional area had its own characteristics. The sampling sites were set in five functional areas and the concentrations of PAEs in the water at each sampling site are shown in Figure 1 and Table S4. The concentrations of seven typical PAEs were detected, including DMP, DEP, BBP, DBP, DEHP, DOP and DIBP. The results showed that DMP, DEP, BBP and DOP were not detected at all sampling sites, while DBP, DEHP and DIBP were 100% detected. It can be seen from Table S4 that the exposure concentrations of DBP and DEHP were relatively high. The highest concentration of ∑_3_PAEs was at sampling site 22 at a concentration of 1.3 μg/L. In addition, the concentration of ∑_3_PAEs at sampling sites in the inflow rivers and inflow area were relatively high. The main reason might be that a large number of high-water-consuming and heavily polluting industrial enterprises such as chemical fiber, papermaking, and batteries were gathered in the upper reaches of the rivers, resulting in a lot of industrial sewage. The inflow rivers mixed with the water in the inflow area, which reduced the pollution concentration of ∑_3_PAEs. Due to the influence of human activities, including tourism, domestic sewage discharge, and unreasonable application of pesticides and fertilizers, the ∑_3_PAEs concentration of aquaculture, living areas and tourist areas was secondary. The lowest concentration was located in the primitive area, because it is an undeveloped area with less human intervention. Therefore, in general, the pollution of PAEs in the inflow area was the most serious. Meanwhile, DBP and DEHP were the most widely used PAEs, which had higher concentration in water [41].

### 3.2. The Ecological Risk Assessments of PAEs in Baiyangdian Lake

The ALCs of typical PAEs in this study are listed in Table 1. These values were obtained through SSD, which were constructed based on reproductive toxicity endpoints. Because the reproductive toxicity endpoints were more sensitive than the lethal toxicity effect endpoint, the obtained ALCs of PAEs can avoid the adverse effect to aquatic organisms due to long-term exposure at low concentration and can better protect aquatic organisms.

**Figure 3:**
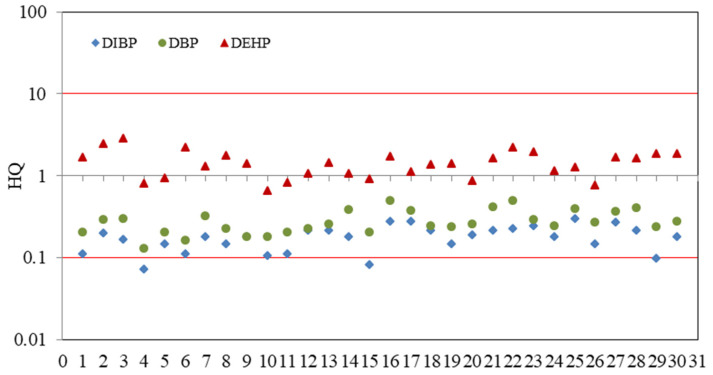
Ecological risk assessments of ∑_3_PAEs in Baiyangdian Lake.

The HQs of DIBP, DBP and DEHP are shown in Table S4 and Figure 3. Obviously, the HQs of DBP at all sampling sites were in the range of 0.1–1.0, indicating that DBP posed a low ecological risk in Baiyangdian Lake. Moreover, in view of the DIBP, the HQs at about 86.7% of the sampling sites were also in the range of 0.1–1.0, showing that 86.7% of the sampling sites in Baiyangdian Lake had low risk, and the HQs at other sampling sites were less than 0.1, so the rest of the sampling had no risk. However, the HQs of DEHP were greater than 1.1 at most sampling sites, and the HQs of the remaining sampling sites were also in the range of 0.1–1.0, manifesting that DEHP had low or medium ecological risk. In contrast, the ecological risks of DBP and DIBP were much lower than DEHP, but their potential ecological risk cannot be ignored.

**Figure 4:**
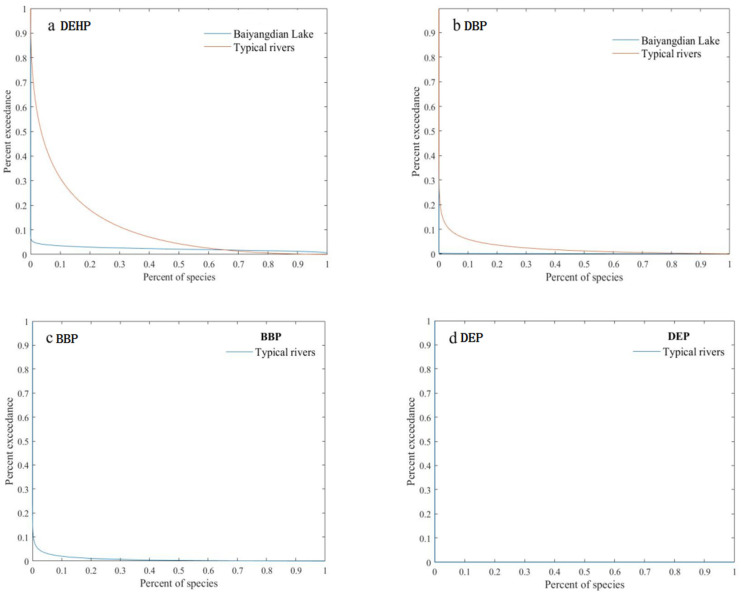
Joint probability curves for ecological risks of PAEs. (**a**) Joint probability curve of DEHP; (**b**) Joint probability curve of DBP; (**c**) Joint probability curve of BBP; (**d**) Joint probability curve of DEP).

Compared with the HQ method, the PERA method can better describe the ecological risks of PAEs. JPC is one of the commonly used risk assessment methods in the process of the PERA method. The results of the PERA method in Baiyangdian Lake are shown in Figure 4a,b. It can be seen from the figure that the JPC of DBP was closer to the *x*-axis than that of DEHP, indicating that the potential ecological risk of DBP was higher than that of DEHP. The results of PERAs in Baiyangdian Lake showed that the probabilities of DEHP and DBP affecting 5% aquatic organisms by JPCs were 3.97% and 0.20%, respectively.

### 3.3. The Ecological Risk Assessments of PAEs in Typical Rivers

In this study, the exposure concentrations of DBP, DEHP, DMP, DEP, BBP, DOP and DIBP in different typical rivers in China were collected from the published literature and listed in Table 2. The data were the average values of PAEs exposure concentrations except for the Zhenjiang section of the Yangtze River, which were the maximum exposure concentrations of PAEs.

**Figure 5:**
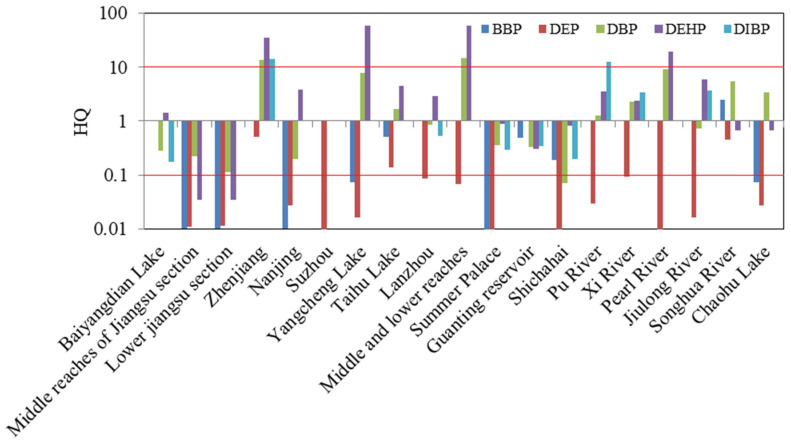
The preliminary ecological risks of ∑_5_PAEs in typical rivers in China (in water).

The ecological risk assessments of PAEs in typical rivers in China were carried out by the HQ method. The results are shown in Table S5 and Figure 5. For the BBP, the HQs of Taihu Lake, Guanting Reservoir, Shichahai and Songhua River were in the range of 0.1–1.0, so these rivers had low risk, and the rest had no risk because their HQs were less than 0.1. Similarly, DEP had low risk in Taihu Lake and Songhua River; in addition, it also had a low risk in Zhenjiang, and its HQ was slightly higher than that of Taihu Lake and Songhua River. The risk posed by DBP was high at the Zhenjiang section of the Yangtze River and the middle and lower reaches of the Yellow River, while the rest had medium or low risk. In view of DEHP, about 21.1% of rivers had high risk, 10.5% had no risk, and others had medium or low risk. DIBP posed high risk in the Zhenjiang section of the Yangtze River and Pu River, a tributary of Liao River, and it posed medium risk in the Xi River and Jiulong River, while the rest had medium or low risk. DEHP mainly came from plastics and heavy chemical industry as well as domestic waste. DBP was widely used in cosmetics and personal care products, while DIBP has been widely used as a substitute for DBP in recent years. Therefore, with the extensive use of plastics and the development of urban industrialization, a large number of DEHP, DBP and DIBP were produced. The exposure concentrations of DEHP, DBP and DIBP in freshwater in China were higher due to surface runoff or atmospheric wet deposition.

The PERA method was used to evaluate the ecological risks of PAEs in typical rivers, and the JPCs are shown in Figure 4. It can be seen from the figure that the JPCs of BBP and DEP were closer to the *x*-axis than the DEHP and DBP, indicating that the potential ecological risks of DEHP and DBP were higher than BBP and DEP, which also indicated that aquatic organisms were affected by DEHP and DBP obviously, and they led to a higher probability of ecological risk. The results of PERAs in typical rivers in China showed that the probabilities of DEHP, DBP and BBP affecting 5% aquatic organisms by JPCs were 44.2%, 8.6% and 3.0%, respectively, while DEP was 0, meaning they affect less than 5% of the aquatic organisms.

### 3.4. The Ecological Risk Assessments of PAEs in Sediments

SQCs derived by the equilibrium partitioning method based on the ALCs of PAEs and the exposure data of DBP, DEHP, DMP, DEP, BBP, DOP and DIBP in sediments are listed in Table 1 and Table 3 respectively. The exposure data were all the maximum values of PAEs.

For example, the content of DMP in sediments from Taihu Lake Basin was as high as 3.50 μg/gdw, which was hundreds of times higher compared with Baiyangdian Lake. The maximum content of DMP, DEP and DOP appeared in Taihu Lake. The concentrations of DMP and DEP were similar and in an order of magnitude, while the concentration of DOP is relatively high, reaching 16.2 μg/gdw.

**Figure 6:**
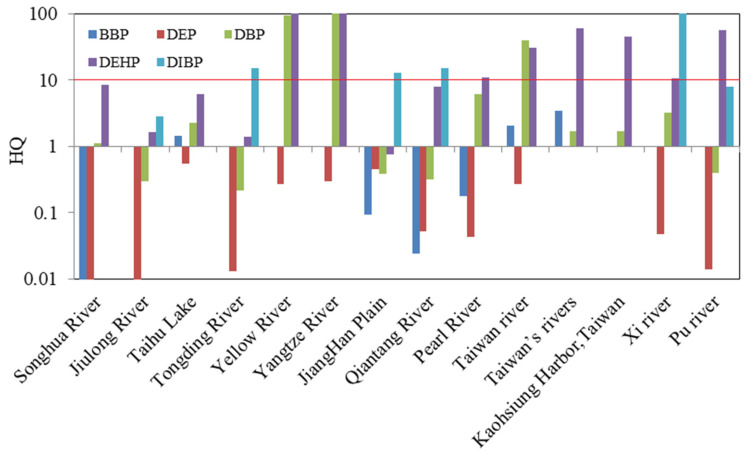
The preliminary ecological risks of ∑_5_PAEs in typical rivers in China (in sediments).

The ecological risk assessments of PAEs in sediments from typical rivers in China were carried out only by the HQ method. The HQs of each river are shown in Table S6. Among them, BBP pose low risk in sediments from Taihu Lake, and its HQ was1.47, which was inconsistent with previous study [45]. The reason may be that the ALC obtained in this study based on the reproductive toxicity endpoint was stricter than that based on the non-lethal toxicity endpoint in previous studies. Meanwhile, there was a medium risk in sediments from Taiwan’s rivers, and the other rivers had low or no risk. As a result of the lg$K_{\mathrm{ow}}$ of DEP being 2.38, less than 3, and its hydrophobicity being weak, it is not easy to be adsorbed in sediment, so its risk to benthic organisms is not considered. For the DBP, about 35.7% of the rivers posed low risk, 42.9% of the rivers posed medium risk, and only 21.4% posed high risk. Risk posed by DEHP was high at 57.1% rivers, except for the JiangHan Plain, while other rivers posed a medium risk. DIBP posed a low risk in most rivers, and their HQs were within the range of 0.1–1, and only the Kaohsiung Harbor in Taiwan posed a medium risk. The preliminary ecological risks of ∑_5_PAEs in sediments from typical rivers in China by HQ method are shown in Figure 6.

## 4. Discussion

Compared with other typical rivers in Table 2, only DBP, DEHP and DIBP were detected in Baiyangdian Lake. Among them, the exposure concentration of DBP in Baiyangdian Lake was much lower than that in the Zhenjiang section of the Yangtze River basin, Yangcheng Lake, the middle and lower reaches of the Yellow River and the Pearl River, and there was an order of magnitude difference. It was equivalent to the exposure concentrations in the middle and lower reaches of the Jiangsu section of the Yangtze River, Nanjing section of the Yangtze River, Summer Palace and Guanting Reservoir. Only Shichahai exposure concentration was lower than the DBP in Baiyangdian Lake. In conclusion, the exposure concentration of DBP in Baiyangdian Lake was at a medium to low level compared with other typical rivers in China. Similar to DBP, the exposure concentration of DEHP in Baiyangdian Lake was also much lower than that in the Zhenjiang section of the Yangtze River, Yangcheng Lake and the middle and lower reaches of the Yellow River. In addition, the exposure concentration was similar to those in the Lanzhou section of the Yellow River, Summer Palace, Shichahai and Xi River. However, different from DBP, the exposure concentrations of DEHP in the middle and lower reaches of the Jiangsu section of the Yangtze River, Guanting Reservoir and Chao Lake were lower than that in Baiyangdian Lake, indicating that the concentration of DEHP in Baiyangdian Lake was at a medium to low level. Therefore, the pollution degree of PAEs is at a lower level in Baiyangdian Lake compared with other domestic water bodies, and the main pollutant is DEHP, which is followed by DBP. This result is consistent with the study of Taihu, which shows that DBP and DEHP were the most abundant PAE congeners in surface water [45], and DBP and DEHP have a certain homology. Although the concentration of DIBP was detected in Baiyangdian Lake, the concentration was very low.

For the rivers near or passing through industrial cities, such as the Zhenjiang section of the Yangtze River and Pu River, a tributary of the Liao River, the exposure concentrations of DIBP were relatively high, while in other rivers they were relatively low, and it was even not detected in a few rivers. A large number of DBP, DEHP and DIBP entered the water with surface runoff or atmospheric wet deposition. Because of their strong hydrophobicity, they often combine with particulate matter, settle in the bottom sediment and accumulate continuously, resulting in the high content of DBP, DEHP and DIBP in typical rivers sediments in China. The highest concentrations of DBP and DEHP occurred in the Yangtze River and the Yellow River, respectively, and their concentrations can be as high as several hundred μg/gdw. DIBP has also been widely used as a substitute for DBP in recent years, which had increased the content in sediment. According to previous studies, the highest concentration appears in the Xihe River [59]. Although the production and consumption of these PAEs in daily life were very low, due to the developed agriculture around Taihu Lake, a large amount of agricultural runoff flowed into the lake, resulting in the high content of PAEs in sediment.

The ecological risk assessments of DEHP and DBP in Baiyangdian Lake were carried out by HQ and PERA. The results of the two methods were consistent, indicating that DEHP and DBP in Baiyangdian Lake water had potential ecological risk to aquatic organisms and regional ecosystem. However, the ecological risks of DEHP and DBP are evaluated based on Chinese environmental quality standards for surface water (the WQS of DEHP was 8 μg/L and DBP was 3 μg/L), the range of HQs were 0.0240–0.104 and 0.0400–0.153, respectively, indicating that there were no ecological risks of DEHP and DBP in most sampling sites of Baiyangdian Lake, and only a few sampling sites had low risk. Therefore, according to the Chinese current environmental quality standards, the ecological risks of PAEs in some rivers would be underestimated. The HQ method and PERA method were adopted for DEHP and DBP, and the results were consistent. In other words, both DEHP and DBP posed potential ecological risk in typical rivers. It is consistent with the results of the Liao River, and the study show that the ecological risk of DEHP in Liao River should be paid more attention [59]. So, it was necessary to strengthen the management and control of these two PAEs. DEHP values of 80.95 of the sediment samples of Songhua river exceeded the low effects range [32], and DEHP posed high ecological risk in typical river sediments, which was followed by DBP and DIBP. These studies have similar results, indicating that PAEs are potentially harmful to the aquatic environment.

## 5. Conclusions

The exposure concentrations of typical PAEs in Baiyangdian Lake were measured by field sampling in this study, and the results showed that the detection rates of DBP, DEHP and DIBP were higher. The PAEs exposure data in typical rivers and their sediments in China were obtained through published literature. Subsequently, according to the ALCs of typical PAEs derived from reproduction toxicity endpoints, the ecological risks of typical PAEs in Baiyangdian Lake water and typical rivers were evaluated by the HQ method and PERA method. Based on SQCs, which were derived using the equilibrium partitioning method by the ALCs of PAEs, only the HQ method was used to evaluate the ecological risks of typical PAEs in sediments from typical rivers. The results of the HQ method and PERA method showed that in terms of water, DBP and DIBP posed low risk in Baiyangdian Lake, but the risk of DEHP was slightly higher and cannot be ignored. Different from Baiyangdian Lake, in addition to the high ecological risk of DEHP in typical rivers in China, the risks of DBP and DIBP also cannot be ignored. Furthermore, the HQ method was used to evaluate the sediment risk about PAEs, and the results showed that DEHP posed a high ecological risk in typical rivers sediments, which was followed by DBP and DIBP. In conclusion, the ALCs of DEHP and DBP obtained in this study can be used to evaluate the ecological risks of Baiyangdian Lake and typical rivers more accurately. Meanwhile, in this study, the pollution status of PAEs in Baiyangdian was investigated, the pollution level, spatial distribution characteristics and sources of PAEs in typical lake were explored, and the ecological risks under their exposure levels were measured, so as to provide technical support for pollution prevention and environmental risk management in typical lakes worldwide.

## Figures and Tables

**Table 1:** Parameters of SSDs for four PAEs (exclude DIBP).

PAEs	N	Mean	${\mathrm{HC}}_{5}$ (μg/L)	ALC (μg/L)	SQC (μg/g dw)
DEHP	12	223.44	0.87000	0.29000	0.77604
DBP	14	3009.4	2.8100	0.93667	0.76115
BBP	7	7427.7	2.9700	0.99000	0.88773
DEP	8	10,862	15.830	5.2767	4.1376
DIBP	-	-	-	0.90000	0.050000

-: No data. N: Number.

**Table 2:** Exposure data of PAEs in typical rivers in China (μg/L).

Rivers	Sites	Concentration (μg/L)							
		DBP	DEHP	DMP	DEP	BBP	DOP	DIBP	Reference
Baiyangdian Lake		0.26	0.42	ND	ND	ND	ND	0.16	This study
Pearl River		8.5	5.6	2.4	0.046	ND	ND	ND	[42]
Jiulong River		0.67	1.7	0.088	0.085	ND	ND	3.4	[34]
Songhua River		5.1	0.20	2.5	2.4	2.5	2.4	ND	[43]
Chaohu Lake		3.2	0.20	0.42	0.14	0.071	0.035	ND	[44]
Yangtze River	Middle reaches of Jiangsu section	0.21	0.010	0.025	0.057	0.010	0.010	ND	[45]
	Lower jiangsu section	0.11	0.010	0.013	0.060	0.010	0.010	ND	[45]
	Zhenjiang	13	10	1.5	2.6	ND	1.1	13	[46]
	Nanjing	0.19	1.1	0.010	0.14	0.010	0.020	ND	[45]
	Suzhou	ND	ND	0.015	0.012	ND	0.034	ND	[47]
	Yangcheng Lake	7.2	17	0.13	0.086	0.072	0.34	ND	[47]
	Taihu Lake	1.6	1.3	0.71	0.72	0.50	0.16	ND	[48]
Yellow River	Lanzhou	0.80	0.83	0.64	0.46	ND	0.0020	0.48	[49]
	Middle and lower reaches	14	17	0.24	0.36	ND	1.9	ND	[9]
Haihe River	Summer Palace	0.34	0.26	0.062	0.0060	0.0060	0.019	0.26	[50]
	Guanting reservoir	0.30	0.087	0.056	ND	0.48	0.017	0.31	[50]
	Shichahai	0.066	0.24	0.081	0.0090	0.19	0.019	0.18	[50]
Liao River	Pu River	1.2	1.0	0.66	0.16	ND	ND	11	[51]
	Xi River	2.2	0.70	0.46	0.49	ND	ND	3.1	[51]

ND: Not Detected.

**Table 3:** Part of PAEs in sediments from typical rivers in China (μg/gdw).

Rivers	Concentration (μg/L)							
	DMP	DEP	DBP	BBP	DEHP	DOP	DIBP	Reference
Songhua River	0.00300	0.0170	0.852	0.00500	6.56	0.0420	ND	[32]
Jiulong River	0.00400	0.00600	0.230	ND	1.28	ND	0.140	[34]
Taihu Lake	3.50	2.29	1.75	1.30	4.77	16.2	ND	[45]
Tongding River	0.0210	0.0540	0.165	ND	1.09	0.0200	0.750	[52]
Yellow River	1.04	1.12	72.2	ND	258	ND	ND	[9]
Yangtze River	2.24	1.24	246	ND	221	ND	ND	[53]
JiangHan Plain	0.238	1.87	0.290	0.0820	0.596	ND	0.639	[54]
Qiantang River	0.179	0.218	0.241	0.0210	6.24	0.0190	0.769	[55]
Pearl River	1.75	0.180	4.66	0.160	8.53	0.310	ND	[49]
Taiwan river	ND	1.10	30.3	1.80	23.9	ND	ND	[56]
Taiwan’s rivers	ND	ND	1.30	3.10	46.5	ND	ND	[57]
Kaohsiung Harbor, Taiwan	ND	ND	1.31	ND	34.8	0.600	ND	[58]
Xi river	0.266	0.197	2.43	ND	8.30	4.35	11.2	[2]
Pu river	0.0530	0.0600	0.304	ND	44.5	1.47	0.404	[2]

ND: Not Detected.

## Data Availability

Not applicable.

## Supplementary Materials

The following supporting information can be downloaded at: https://www.mdpi.com/article/10.3390/toxics11020180/s1, Table S1: Characterization of 7 PAEs investigated; Table S2: Reproductive toxicity data used to construct SSDs of DEHP, BBP, DEP, and DBP; Table S3: The average chronic toxicity value and cumulative frequency of DEHP, BBP, DEP, and DBP; Table S4: Exposure concentrations and HQ values of ∑3 PAEs in Baiyangdian Lake; Table S5: Summary of HQ values in water; Table S6: Summary of HQ values in sediments.

## References

[1] Zhang Z.-M., Zhang H.-H., Zhang J., Wang Q.-W., Yang G.-P. (2018). Occurrence, distribution, and ecological risks of phthalate esters in the seawater and sediment of Changjiang River Estuary and its adjacent area. Sci. Total Environ..

[2] Li B., Liu R., Gao H., Tan R., Zeng P., Song Y. (2016). Spatial distribution and ecological risk assessment of phthalic acid esters and phenols in surface sediment from urban rivers in Northeast China. Environ. Pollut..

[3] Janjua N.R., Mortensen G.K., Andersson A.-M., Kongshoj B., Skakkebæk N.E., Wulf H.C. (2007). Systemic Uptake of Diethyl Phthalate, Dibutyl Phthalate, and Butyl Paraben Following Whole-Body Topical Application and Reproductive and Thyroid Hormone Levels in Humans. Environ. Sci. Technol..

[4] Prasad B., Prasad K.S., Dave H., Das A., Asodariya G., Talati N., Swain S., Kapse S. (2022). Cumulative human exposure and environmental occurrence of phthalate esters: A global perspective. Environ. Res..

[5] Hajiouni S., Mohammadi A., Ramavandi B., Arfaeinia H., De-La-Torre G.E., Tekle-Röttering A., Dobaradaran S. (2021). Occurrence of microplastics and phthalate esters in urban runoff: A focus on the Persian Gulf coastline. Sci. Total Environ..

[6] Nikolopoulou V., Alygizakis N.A., Nika M.C., Oswaldova M., Oswald P., Kostakis M., Slobodnik J. (2022). Screening of legacy and emerging substances in surface water, sediment, biota and groundwater samples collected in the Siverskyi Donets River Basin employing wide-scope target and suspect screening. Sci. Total Environ..

[7] Zhang D., Liu W., Wang S., Zhao J., Xu S., Yao H., Wang H., Bai L., Wang Y., Gu H. (2021). Risk assessments of emerging contaminants in various waters and changes of microbial diversity in sediments from Yangtze River chemical contiguous zone, Eastern China. Sci. Total Environ..

[8] Chai X.L., Ji R., Wu J., Tong H.H., Zhao Y.C. (2010). Abiotic association of PAEswith humic substances and its influence on the fate of PAEs in landfillleachate. Chemosphere.

[9] Sha Y.J., Xia X.H., Yang Z.F., Huang G.H. (2007). Distribution of PAEs in the middle and lower reaches of the YellowRiver, China. Environ. Monit. Assess..

[10] Wang P., Wang S.L., Fan C.Q. (2008). Atmospheric distribution of particulate and gasphase phthalic esters (PAEs) in a Metropolitan City, Nanjing, East China. Chemosphere.

[11] Qiu Y.-W., Wang D.-X., Zhang G. (2020). Assessment of persistent organic pollutants (POPs) in sediments of the Eastern Indian Ocean. Sci. Total Environ..

[12] Le T.M., Nguyen H.M.N., Nguyen V.K., Nguyen A.V., Vu N.D., Yen N.T.H., Hoang A.Q., Minh T.B., Kannan K., Tran T.M. (2021). Profiles of phthalic acid esters (PAEs) in bottled water, tap water, lake water, and wastewater samples collected from Hanoi, Vietnam. Sci. Total Environ..

[13] Selvaraj K.K., Sundaramoorthy G., Ravichandran P.K., Girijan G.K., Sampath S., Ramaswamy B.R. (2015). Phthalate esters in water and sediments of the Kaveri River, India: Environmental levels and ecotoxicological evaluations. Environ. Geochem. Health.

[14] Liu N., Wang Y., Yang Q., Lv Y., Jin X., Giesy J.P., Johnson A.C. (2016). Probabilistic assessment of risks of diethylhexyl phthalate (DEHP) in surface waters of China on reproduction of fish. Environ. Pollut..

[15] Zhang Q.Q., Ying G.G., Pan C.G., Liu Y.S., Zhao J.L. (2015). Comprehensive Evaluation of Antibiotics Emi-ssion and Fate in the River Basins of China: Source Analysis, Multimedia Modeling, and Linkage to Bacterial Resistance. Environ. Sci. Technol..

[16] Kamrin M.A. (2009). Phthalate risks, phthalate regulation, and public health: A review. Toxicol. Environ. Health B Crit. Rev..

[17] Yuan S.-Y., Huang I.-C., Chang B.-V. (2010). Biodegradation of dibutyl phthalate and di-(2-ethylhexyl) phthalate and microbial community changes in mangrove sediment. J. Hazard. Mater..

[18] US EPA (2014). Priority Pollutants. http://water.epa.gov/scitech/methods/cwa/pollutants.cfm.

[19] (2022). Environmental Quality Standard for Surface Water.

[20] Wu F., Meng W., Zhao X., Li H., Zhang R., Cao Y., Liao H. (2010). China Embarking on Development of its Own National Water Quality Criteria System. Environ. Sci. Technol..

[21] Wheeler J.R., Grist E.P., Leung K.M., Morritt D., Crane M. (2002). Species sensitivity distributions: Data and model choice. Mar. Pollut. Bull..

[22] Jiang M., Li Y., Zhang B., Zhou A., Zhu Y., Li J., Xu S. (2018). Urinary concentrations of phthalate metab-olites associated with changes in clinical hemostatic and hematologic parameters in pr-egnant women. Environ. Int..

[23] Zhang B., Zhang T., Duan Y., Zhao Z., Huang X., Bai X., Xie L., He Y., Ouyang J., Yang Y. (2019). Human exposure to phthalate esters associated with e-waste dismantling: Exposure levels, sources, and risk assessment. Environ. Int..

[24] Caldwell D.J., Mastrocco F., Hutchinson T.H., Länge R., Heijerick D., Janssen C., Anderson P.D., Sumpter J.P. (2008). Derivation of an Aquatic Predicted No-Effect Concentration for the Synthetic Hormone, 17α-Ethinyl Estradiol. Environ. Sci. Technol..

[25] Jin X., Wang Y., Jin W., Rao K., Giesy J.P., Hollert H., Richardson K.L., Wang Z. (2013). Ecological Risk of Nonylphenol in China Surface Waters Based on Reproductive Fitness. Environ. Sci. Technol..

[26] Martino-Andrade A.J., Chahoud I. (2010). Reproductive toxicity of phthalate esters. Mol. Nutr. Food Res..

[27] US EPA (1998). Guidelines for Ecological Risk assessment.Ecological Risk Assessment Step 2.

[28] Solomon K., Giesy J., Jones P. (2000). Probabilistic risk assessment of agrochemicals in the environment. Crop. Prot..

[29] Wang X., Tao S., Dawson R., Xu F. (2002). Characterizing and comparing risks of polycyclic aromatic hydrocarbons in a Tianjin wastewater-irrigated area. Environ. Res..

[30] Giesy J.P., Solomon K.R., Coats J.R., Dixon K.R., Giddings J.M., Kenaga E.E. (1999). Chlorpyrifos: Ecological risk assessment in North American aquatic environments. Rev. Environ. Contam. Toxicol..

[31] Paluselli A., Kim S.K. (2020). Horizontal and vertical distribution of phthalates acid ester (PAEs) in seawater and sediment of East China Sea and Korean South Sea: Traces of plastic debris?. Mar. Pollut. Bull..

[32] Yan Z., Pan J., Gao F., An Z., Liu H., Huang Y., Wang X. (2019). Seawater quality criteria derivation and ecological risk assessment for oil pollution in China. Mar. Pollut. Bull..

[33] MEP (2022). Ministry of Ecology and Environment of the People’s Republic of China, HJ 831- 2022: Technical Guideline for Deriving Water Quality Criteria for the Protection of Freshwater Aquatic Organisms.

[34] Li R., Liang J., Gong Z., Zhang N., Duan H. (2017). Occurrence, spatial distribution, historical trend and ecological risk of phthalate esters in the Jiulong River, Southeast China. Sci. Total Environ..

[35] Jin X., Wang Y., Giesy J.P., Richardson K.L., Wang Z. (2014). Development of aquatic life criteria in China: Viewpoint on the challenge. Environ. Sci. Pollut. Res..

[36] Lemly A. (1996). Evaluation of the Hazard Quotient Method for Risk Assessment of Selenium. Ecotoxicol. Environ. Saf..

[37] (2003). European Commission Technical Guidance on Risk Assessment in Support of Commission Directive 93/67/EEC on Risk Assessment for New Notified Substances Commission Regulation (EC) No 1488/94[R].

[38] Shi R., Yang C., Su R., Jin J., Chen Y., Liu H., Giesy J.P., Yu H. (2014). Weighted species sensitivity distribution method to derive site-specific quality criteria for copper in Tai Lake, China. Environ. Sci. Pollut. Res..

[39] Wang Y., Zhang L., Meng F., Zhou Y., Jin X., Giesy J.P., Liu F. (2014). Improvement on species sensitivity distribution methods for deriving site-specific water quality criteria. Environ. Sci. Pollut. Res..

[40] Xia L.L., Liu R.Z., Zao Y.W. (2012). Correlation analysis of landscape pattern and water quality in Baiyangdian watershed. Procedia Environ. Sci..

[41] Jin D., Kong X., Li Y., Bai Z., Zhuang G., Zhuang X., Deng Y. (2015). Biodegradatio-n of di-n-Butyl Phthalate by Achromobacter sp Isolated from Rural Domestic Wastewater. Int. J. Environ. Res. Health.

[42] Li X., Yin P., Zhao L. (2016). Phthalate esters in water and surface sediments of the Pearl River Estuary: Distribution, ecological, and human health risks. Environ. Sci. Pollut. Res..

[43] Gao D., Li Z., Wen Z., Ren N. (2014). Occurrence and fate of phthalate esters in full-scale domestic wastewater treatment plants and their impact on receiving waters along the Songhua River in China. Chemosphere.

[44] He W., Qin N., Kong X., Liu W., He Q., Ouyang H., Yang C., Jiang Y., Wang Q., Yang B. (2013). Spatio-temporal distributions and the ecological and health risks of phthalate esters (PAEs) in the surface water of a large, shallow Chinese lake. Sci. Total Environ..

[45] He H., Hu G.J., Sun C., Chen S.L., Yang M.N., Li J., Zhao Y., Wang H. (2011). Trace analysis of persistent toxic substances in the main stream of Jiangsu section of the Yangtze River, China. Environ. Sci. Pollut. Res..

[46] Chen H., Mao W., Shen Y., Feng W., Mao G., Zhao T., Wu X. (2019). Distribution, source, and environmental risk assessment of phthalate esters (PAEs) in water, suspended particulate matter, and sediment of a typical Yangtze River Delta City, China. Environ. Sci. Pollut. Res. Int..

[47] Zhang L., Dong L., Ren L., Shi S., Zhou L., Zhang T., Huang Y. (2012). Concentration and source identification of polycyclic aromatic hydrocarbons and phthalic acid esters in the surface water of the Yangtze River Delta, China. J. Environ. Sci..

[48] Gao X., Li J., Wang X., Zhou J., Fan B., Li W., Liu Z. (2019). Exposure and ecological risk of phthalate esters in the Taihu Lake basin, China. Ecotoxicol. Environ. Saf..

[49] Zhao X., Shen J.M., Zhang H., Li X., Chen Z.L., Wang X.C. (2020). The occurrence and spatial distribution of phthalate esters (PAEs) in the Lanzhou section of the Yellow River. Environ. Sci. Pollut. Res..

[50] Zheng X., Zhang B.-T., Teng Y. (2014). Distribution of phthalate acid esters in lakes of Beijing and its relationship with anthropogenic activities. Sci. Total Environ..

[51] Li B., Hu X., Liu R., Zeng P., Song Y. (2015). Occurrence and distribution of pht-halic acid esters and phenols in Hun River Watersheds. Environ. Earth Sci..

[52] Wang X.T., Ma L.L., Sun Y.Z., Xu X.B. (2006). Phthalate esters in sediments from Guanting Reservoir and the Yongding River, Beijing, People’s Republic of China. Bull. Environ. Contam. Toxicol..

[53] Fan W., Xinghui X., Yujuan S. (2008). Distribution of phthalic acid esters in Wuhan section of the Yangtze River, China. J. Hazard. Mater..

[54] Liu H., Liang H., Liang Y., Zhang D., Wang C., Cai H., Shvartsev S.L. (2010). Distribution of phthalate esters in alluvial sediment: A case study at JiangHan Plain, Central China. Chemosphere.

[55] Sun J., Huang J., Zhang A., Liu W., Cheng W. (2013). Occurrence of phthalate esters in sediments in Qiantang River, China and inference with urbanization and river flow regime. J. Hazard. Mater..

[56] Yuan S.Y., Liu C., Liao C.S., Chang B.V. (2002). Occurrence and microbial degrada-tion of phthalate esters in Taiwan river sediments. Chemosphere.

[57] Huang P.-C., Tien C.-J., Sun Y.-M., Hsieh C.-Y., Lee C.-C. (2008). Occurrence of phthalates in sediment and biota: Relationship to aquatic factors and the biota-sediment accumulation factor. Chemosphere.

[58] Chen C.W., Chen C.F., Dong C.D. (2013). Distribution of Phthalate Esters in Sediments of Kaohsiung Harbor, Taiwan. Taylor Fr. Group..

[59] Zheng X., Yan Z., Liu P., Li H., Zhou J., Wang Y., Fan J., Liu Z. (2018). Derivation of aquatic life criteria for four phthalate esters and their ecological risk assessment in Liao River. Chemosphere.

