# Supplementary material for: Risk Assessment of Phthalate Esters in Baiyangdian Lake and Typical Rivers in China
Source: Toxics. 2023 Feb 15;11(2):180. doi: 10.3390/toxics11020180 (PMC9962510; doi:10.3390/toxics11020180)
Supplement: Supplementary file 1 [file toxics-11-00180-s001.zip › toxics-2174122-supplementary.pdf]

Supplementary Material

Risk Assessment of Phthalate Esters in Baiyangdian Lake and Typical Rivers in China

Yin Hou [1,2], Mengchen Tu [1], Cheng Li [3], Xinyu Liu [1], Chao Wei [1], Jing Wang [1,2], Xin Zheng [1,*] and Yihong Wu [3,*]

[1] State Key Laboratory of Environmental Criteria and Risk Assessment, Chinese Research Academy of Environmental Sciences, Beijing 100012, China

[2] College of Marine Ecology and Environment, Shanghai Ocean University, Shanghai 201306, China

[3] Institute of Green Development, Hebei provincial Academy of Environmental Sciences, Shijiazhuang, 050037, China

* Correspondence: zhengxin@craes.org.cn (X.Z.); yh_wuhb@126.com (Y.W.)

Table S1. Characterization of 7 PAEs investigated

**Phthalate esters**	**Acronym**	**CAS**	**Alkyl-chain length**	**Molecular formula**	**Molecular weight**	**Solubility in water (mg/L)**	**LgK_{ow}**	**K_{oc} (L/kg)**
Dimethyl phthalate	DMP	131-11-3	1	$C_{10}H_{10}O_4$	194	2014	1.61	40
Diethyl phthalate	DEP	84-66-2	2	$C_{12}H_{14}O_4$	222	287	2.24	69
Diisobutyl phthalate	DIBP	84-69-5	3	$C_{16}H_{22}O_4$	278	0.00	4.11	-
Dibutyl phthalate	DBP	84-74-2	4	$C_{16}H_{22}O_4$	278	2.35	4.27	1380
Butyl benzyl phthalate	BBP	85-68-7	4	$C_{19}H_{20}O_4$	312	0.95	4.70	5248
Di-n-octyl phthalate	DOP	117-84-0	8	$C_{24}H_{38}O_4$	390	0.00	8.18	23988
Bis(2-ethylhexyl) phthalate	DEHP	117-81-7	8	$C_{20}H_{14}O_4$	390	0.00	7.73	87096

-: No data

Table S2. Reproductive toxicity data used to construct SSDs of DEHP, BBP, DEP, and DBP

Phthalate esters	Test species	Duration (days)	Endpoint	Measurement	Con. (μg/L)	Reference
DEHP	*Eurytemora affinis*	21	NOEC	REP	109	Forget-Leray, et al, 2005
	Oryzias melastigma	180	LOEC	MPH	100	Ye, et al, 2014
	Oryzias latipes	91	NOEC	REP	1	Kim et al, 2002
	Salvelinus fontinalis	150	NOEC	MPH	3.7	Mayer et al,1977
	Daphnia magna	21	NOEC	REP	640	Adams and Heidolph,1985
	Gobiocypris rarus	21	NOEC	MPH	3.6	Wang, et al, 2013
	Danio rerio	21	LOEC	REP	20	Carnevali, et al, 2010
	Pimephales promelas	28	NOEC	MPH	12	Crago and Klaper, 2012
	Poecilia reticulata	28	NOEC	MPH	320	Adema and Zwart, 1984
	Oncorhynchus mykiss	42	NOEC	GRO	502	Spehar, R.L., 1986
	Pseudokirchneriella subcapitata	5	EC_{50}	POP	960	Richter,1982

Phthalate esters	Test species	Duration (days)	Endpoint	Measurement	Con. (μg/L)	Reference
DBP	*Hydra viridissima*	7	NOEC	POP	10	Ganeshakumar, M., 2009
	Oncorhynchus mykiss	99	NOEC	GRO	100	Rhodes, et al, 1995
	Gasterosteus aculeatus	22	NOEC	GRO	35.2	Aoki, et al, 2011
	Melanotaenia fluviatilis	7	NOEC	REP	14	Bhatia, et al, 2014
	Glandirana rugosa	21	NOEC	MPH	278	Ohtani et al., 2000
	Xenopus laevis	266	NOEC	MPH	1000	Higuchi, 2002
	Daphnia magna	21	NOEC	REP	960	Kuhn, et al, 1989
	Danio rerio	95	NOEC	GRO	400	Chen et al., 2015
	Pimephales promelas	14	NOEC	GRO	500	Weston et al., 2009
	Pseudokirchneriella subcapitata	4	NOEC	POP	210	Adams, et al, 1995
	Scenedesmus subspicatus	3	NOEC	POP	500	Scholz, N., 1995
	Chlorella vulgaris	4	EC_{50}	POP	7780	Chi et ai,2006

Phthalate esters	Test species	Duration (days)	Endpoint	Measurement	Con. (μg/L)	Reference
BBP	*Scenedesmus acutus*	3	NOEC	POP	30200	Kuang et al,2003
	Lemna minor	7	LOEC	MPH	5	Huang et al., 2006
	Ceratophyllum demersum	25	NOEC	BIO	150	Li et al., 2006
	Pseudokirchneriella subcapitata	4	NOEC	POP	60	USEPA, 1978
	Daphnia magna	20	NOEC	REP	170	E.G. and G. Bionomics, 1979
	Fundulus heteroclitus	28	NOEC	GRO	100	Kaplan,et al,2013
DEP	*Navicula pelliculosa*	4	EC_{50}	POP	600	Gledhill,1980
	Anacystis aeruginosa	4	EC_{50}	POP	1000	Gledhill,1980
	Ipomoea aquatica	28	NOEC	GRO	50000	Chen,et al,2011
	Pimephales promelas	21	NOEC	FEC	64.6	Hicks, 2008
	Chlamydomonas reinhardtii	3	EC10	POP	1020	Brack, W., and H. Rottler, 1994
	Pseudokirchneriella subcapitata	4	NOEC	POP	3650	Adams, et al, 1995

Phthalate esters	Test species	Duration (days)	Endpoint	Measurement	Con. (μg/L)	Reference
	Daphnia magna	21	NOEC	REP	3800	Kuhn, et al, 1989
	Cyprinus carpio	28	LOEC	MPH	1000	Barse, et al, 2007
	Tetrahymena thermophila	2	NOEC	POP	40000	Pauli, et al, 1993
	Danio rerio	3.8	NOEC	REP	427.2	Xu et al,2013
	Anodonta cygnea	4	EC_{50}	POP	16000	Adams and Gorsuch,1995
	Scenedesmus subspicatus	4	EC_{50}	POP	21000	Kuhn et al,1989

POP: population growth rate, REP: reproduction, GRO: growth, MPH: morphology, BEH: behavior, FEC: fecundity, BIO: Biochemistry.

Table S3. The average chronic toxicity value and cumulative frequency of DEHP, BBP, DEP, and DBP

Phthalate esters	Test species	Endpoint	Con. (μg/L)	CVE (μg/L)	lg(CVE) (μg/L)	R	f	F_R (%)
DEHP	*Oryzias latipes*	NOEC	1	1	0	1	1	7.7
	Gobiocypris rarus	NOEC	3.6	1.897	0.278	2	1	15.4

Phthalate esters	Test species	Endpoint	Con. (μg/L)	CVE (μg/L)	lg(CVE) (μg/L)	R	f	F_R (%)
	Salvelinus fontinalis	NOEC	3.7	1.924	0.284	3	1	23.1
	Hydra viridissima	NOEC	10	3.162	0.500	4	1	30.8
	Pimephales promelas	NOEC	12	3.464	0.540	5	1	38.5
	Danio rerio	LOEC	20	4.472	0.651	6	1	46.2
	Oryzias melastigma	LOEC	100	10	1.000	7	1	53.8
	Eurytemora affinis	NOEC	109	10.440	1.019	8	1	61.5
	Poecilia reticulata	NOEC	320	17.889	1.253	9	1	69.2
	Oncorhynchus mykiss	NOEC	502	22.405	1.350	10	1	76.9
	Daphnia magna	NOEC	640	25.298	1.403	11	1	84.6
	Pseudokirchneriella subcapitata	EC_{50}	960	30.984	1.491	12	1	92.3
DBP	*Lemna minor*	LOEC	5	2.236	0.350	1	1	6.7
	Melanotaenia fluviatilis	NOEC	14	3.742	0.573	2	1	13.3

Phthalate esters	Test species	Endpoint	Con. (μg/L)	CVE (μg/L)	lg(CVE) (μg/L)	R	f	F_R (%)
	Gasterosteus aculeatus	NOEC	35.2	5.932	0.773	3	1	20
	Oncorhynchus mykiss	NOEC	100	10	1.000	4	1	26.7
	Ceratophyllum demersum	NOEC	150	12.247	1.088	5	1	33.3
	Pseudokirchneriella subcapitata	NOEC	210	14.491	1.161	6	1	40
	Glandirana rugosa	NOEC	278	16.673	1.222	7	1	46.7
	Danio rerio	NOEC	400	20	1.301	8	1	53.3
	Scenedesmus subspicatus	NOEC	500	22.361	1.350	9	1	60
	Pimephales promelas	NOEC	500	22.361	1.350	10	1	66.7
	Daphnia magna	NOEC	960	30.984	1.491	11	1	73.3
	Xenopus laevis	NOEC	1000	31.623	1.500	12	1	80
	Chlorella vulgaris	EC_{50}	7780	88.204	1.945	13	1	86.7
	Scenedesmus acutus	NOEC	30200	173.781	2.240	14	1	93.3

Phthalate esters	Test species	Endpoint	Con. (μg/L)	CVE (μg/L)	lg(CVE) (μg/L)	R	f	F_R (%)
BBP	*Pseudokirchneriella subcapitata*	NOEC	60	7.476	0.874	1	1	12.5
	Pimephales promelas	NOEC	64.6	8.037	0.905	2	1	25
	Fundulus heteroclitus	NOEC	100	10	1.000	3	1	37.5
	Daphnia magna	NOEC	170	13.038	1.115	4	1	50
	Navicula pelliculosa	EC_{50}	600	24.495	1.389	5	1	62.5
	Anacystis aeruginosa	EC_{50}	1000	31.623	1.500	6	1	75
	Ipomoea aquatica	NOEC	50000	223.607	2.349	7	1	87.5
DEP	*Danio rerio*	NOEC	427.2	20.669	1.315	1	1	11.1
	Cyprinus carpio	LOEC	1000	31.623	1.500	2	1	22.2
	Chlamydomonas reinhardtii	EC_{10}	1020	31.937	1.504	3	1	33.3
	Pseudokirchneriella subcapitata	NOEC	3650	60.415	1.781	4	1	44.4
	Daphnia magna	NOEC	3800	61.644	1.790	5	1	55.5

Phthalate esters	Test species	Endpoint	Con. (μg/L)	CVE (μg/L)	lg(CVE) (μg/L)	R	f	F_R (%)
	Anodonta cygnea	EC_{50}	16000	126.491	2.102	6	1	66.6
	Scenedesmus subspicatus	EC_{50}	21000	144.914	2.161	7	1	77.7
	Tetrahymena thermophila	NOEC	40000	200	2.301	8	1	88.8

Table S4. Exposure concentrations and HQ values of $\sum_3$ PAEs in Baiyangdian Lake

Number	Sampling site	Functional area	DIBP		DBP		DEHP	
			EEC（μg/L）	HQ	EEC（μg/L）	HQ	EEC（μg/L）	HQ
1	Qi li zhuang liu wharf	living area	0.10	0.11	0.19	0.20	0.49	1.69
2	Zao lin zhuang	living area	0.18	0.20	0.27	0.29	0.71	2.45
3	Guang dian zhang zhuang	living area	0.15	0.17	0.28	0.30	0.83	2.86
4	Wang jia sai	tourism area	0.06	0.07	0.12	0.13	0.23	0.79
5	Shao che dian	tourism area	0.13	0.14	0.19	0.20	0.27	0.93

6	Wei hua yuan	tourism area	0.10	0.11	0.15	0.16	0.64	2.21
7	Nan liu zhuang	tourism area	0.16	0.18	0.30	0.32	0.38	1.31
8	Quan tou	living area	0.13	0.14	0.21	0.22	0.51	1.76
9	Xiao bai yang dian duan cun nan	breeding area	0.16	0.18	0.17	0.18	0.41	1.41
10	Dong li zhuang	breeding area	0.09	0.10	0.17	0.18	0.19	0.66
11	Shi hou dian	breeding area	0.10	0.11	0.19	0.20	0.24	0.83
12	Cai pu tai	living area	0.19	0.21	0.21	0.22	0.31	1.07
13	Jin long dian	living area	0.19	0.21	0.24	0.26	0.42	1.45
14	Zhu long he ru dian kou	living area	0.16	0.18	0.36	0.38	0.31	1.07
15	Zao zuo dian	primitive area	0.074	0.08	0.19	0.20	0.26	0.90
16	Bai gou yin ru dian kou	inflow area	0.25	0.28	0.46	0.49	0.50	1.72
17	Jiao cha kou xia	inflow area	0.25	0.28	0.35	0.37	0.32	1.10
18	Xiao li zhen	inflow area	0.19	0.21	0.23	0.24	0.39	1.34

19	Bei pu bu he ru dian kou	inflow area	0.13	0.14	0.22	0.23	0.41	1.41
20	Nan pu bu he ru dian kou	inflow area	0.17	0.19	0.24	0.26	0.25	0.86
21	Cao he ru dian kou	inflow area	0.19	0.21	0.39	0.41	0.47	1.62
22	Cao he	inflow area	0.20	0.22	0.46	0.49	0.64	2.21
23	Qiao nan qiao bei cun	inflow area	0.22	0.24	0.27	0.29	0.56	1.93
24	Liu kou	inflow area	0.16	0.18	0.23	0.24	0.33	1.14
25	Tang he（jiu he dao）	inflow area	0.27	0.30	0.37	0.39	0.37	1.28
26	Tang he（to kou）	inflow area	0.13	0.14	0.25	0.27	0.22	0.76
27	Xiao yi he ru dian kou	inflow area	0.24	0.27	0.34	0.36	0.48	1.66
28	Xiao yi he（pu kou）	inflow area	0.19	0.21	0.38	0.40	0.47	1.62
29	Sai li shang	inflow area	0.09	0.10	0.22	0.23	0.54	1.86
30	Ge cun	inflow area	0.16	0.18	0.26	0.28	0.53	1.83

Table S5. Summary of HQ values in water

Rivers		BBP	DEP	DBP	DEHP	DIBP
Baiyangdian		-	-	0.277	1.434	0.176
Lake	Middle reaches of	0.010	0.011	0.223	0.034	-
Yangtze River	Jiangsu section					
	Lower jiangsu section	0.010	0.011	0.112	0.034	-
	Zhenjiang	-	0.502	13.383	35.207	13.956
	Nanjing	0.010	0.027	0.198	3.872	-
	Suzhou	-	0.002	-	-	-
	Yangcheng Lake	0.073	0.016	7.647	58.421	-
	Taihu Lake	0.505	0.136	1.691	4.448	-
Yellow River	Lanzhou	-	0.086	0.846	2.859	0.537
	Middle and lower	-	0.068	14.936	58.966	-

Haihe River	reaches					
	Summer Palace	0.006	0.001	0.356	0.900	0.294
	Guanting reservoir	0.481	-	0.324	0.300	0.341
Liao River	Lakes Shichahai	0.185	0.002	0.070	0.824	0.198
	Pu River	-	0.030	1.245	3.552	12.444
Pearl River	Xi River	-	0.093	2.287	2.397	3.422
Jiulong River		-	0.009	9.032	19.379	-
Songhua River		-	0.016	0.713	6.000	3.740
Chaohu Lake		2.515	0.445	5.447	0.679	-
		0.072	0.027	3.432	0.679	-

-: No data.

Table S6. Summary of HQ values in sediments

Rivers	BBP	DEP	DBP	DEHP	DIBP
Songhua River	0.006	0.004	1.115	8.454	-
Jiulong River	-	0.001	0.301	1.649	2.800
Taihu Lake	1.466	0.553	2.291	6.147	-
Yongding River	-	0.013	0.216	1.401	15.000
Yellow River	-	0.271	94.437	333.119	-
Yangtze River	-	0.300	321.990	285.309	-
JiangHan Plain	0.092	0.451	0.380	0.768	12.780
Qiantang River	0.024	0.053	0.315	8.040	15.380
Pearl River	0.180	0.043	6.099	10.992	-
Taiwan river	2.029	0.266	39.660	30.799	-
Taiwan's rivers	3.495	-	1.702	59.923	-

Kaohsiung	-	-	1.715	44.845	-
Harbor,Taiwan	-	0.048	3.181	10.696	224.000
Xi river	-	0.014	0.398	57.34	8.080
Pu river					

-: No data
